# Prevalence, Genotype Distribution, and Predictors against HPV Infections Targeted By 2-, 4-, 9-Valent HPV Vaccines among Japanese Males

**DOI:** 10.3390/vaccines8020221

**Published:** 2020-05-14

**Authors:** Yukimasa Matsuzawa, Tadaichi Kitamura, Motofumi Suzuki, Yasuhiro Koyama, Kazuyoshi Shigehara

**Affiliations:** 1 Department of Urology, University of Tokyo Hospital, Bunkyo-ku, Tokyo 113-0033, Japan; ymatsuza@ims.u-tokyo.ac.jp (Y.M.); tadkitamura-tky@umin.ac.jp (T.K.); 2 Japanese Foundation for Sexual Health Medicine, Bunkyo-ku, Tokyo 113-0034, Japan; 3 Department of Urology, Nagareyama Central Hospital, Nagareyama, Chiba 270-0114, Japan; 4 Department of Urology, Asoka Hospital, Koto-ku, Tokyo 135-0002, Japan; yc_ask@hotmail.co.jp; 5 Department of Urology, Faculty of Medicine, Kanazawa University, Kanazawa 920-8641, Japan; kshigehara0415@yahoo.co.jp

**Keywords:** human papillomavirus, HPV vaccine, 2v vaccine, 4v vaccine, 9v vaccine

## Abstract

Objectives: Epidemiological reports of sexual life and human papilloma virus (HPV) infection among Japanese men are scarce, and the necessity of HPV vaccines for males is regarded as a controversial topic in Japan. The objective of this study is to determine the prevalence, genotype distribution, and risk factors against HPV infections targeted by bivalent (2v), quadrivalent (4v), and 9-valent (9v) HPV vaccines among Japanese male patients who visited our urological clinics. Material and Methods: The study population consisted of 798 males aged 20 to 95 years (mean ± standard deviation, 55.4 ± 19.5 years). We collected scraping samples from the glans penis using cotton swabs from all patients for genotyping of HPVs. We compared patients’ characteristics and detected HPV genotypes in order to determine the risk factors against HPV infections. Results: Of 798 participants, 198 participants (198/798; 24.8%) had at least one genotype of any HPV infection. The total number of detected HPV genotypes was 328. Of 328 genotypes, 30% (n = 99; 99/328) were 9v HPV genotypes. Most frequently detected types of high-risk HPV infection were type 52 (n = 40; 40/328; 12.2%). Number of lifetime sex partners (≥21) and present or history of sexually transmitted infections were found to be predictors of any HPV infection with adjusted odds ratios of 3.106 (95% confidence interval (CI), 1.593–6.509) and 1.894 (95% CI, 1.185–3.026), respectively. Age of sex initiation was a predictor of 2v and 4v HPV infections with adjusted odds ratios of 100 (95% CI, 1.013–25.673) and 2.676 (95% CI, 1.037–6.905), respectively. Number of lifetime sex partners (≥21) was a predictor of 9v HPVs with adjusted odds ratios of 2.397 (95% CI, 1.060–5.424). Conclusions: Approximately, a quarter of Japanese male patients who visited urological clinics were exposed to HPV. Moreover, from the perspective of our multivariate logistic regression analysis, some kinds of sexual behavior aggravate the risk of typical HPV genotypes infections.

## 1. Introduction

More than 200 genotypes of human papilloma virus (HPV) have been identified to date [1]. Sexually transmitted HPV genotypes were classified into two groups, low-risk (LR) and high-risk (HR) types [2]. LR HPVs can cause warts around genitals, anus, mouth, and throat. On the other hand, long-lasting infection with HR HPVs can cause anogenital tract cancers, oropharyngeal cancers, and nonmelanoma skin cancers [3]. Each year, approximately 34,800 new cases of HPV-associated cancer were found in the United States and 32,100 cases (92%) of these cancers were attributable to HPV genotypes targeted by the 9-valent (9v) HPV vaccine, with 19,000 cases among females and 13,100 cases among males [4]. Therefore, bivalent (2v) HPV vaccine, which covers HPV 16/18; 4-valent (4v) HPV vaccine, which covers HPV 6/11/16/18; and 9v HPV vaccine, which covers HPV 6/11/16/18/31/33/45/52/58 were recommended for all boys and girls at 11–12 years old in the United States [5]. HPV vaccination has potential not only to prevent HPV-associated diseases in males but also to contribute to herd immunity by the reduction of HPV infection spreading in males and females [6,7,8]. To assess whether existing HPV vaccines are effective for Japanese males, investigating prevalence, genotype distribution, and risk factors among Japanese males against HPV infections targeted by the 2v, 4v, and 9v HPV vaccines is essential. We have already reported the prevalence rate of HPV infection among Japanese males, especially for elderly people [9]. As a sub-analysis of the previous study, we conducted further analysis to determine the genotype distribution and predictors of HPV infections targeted by 2v, 4v, and 9v HPV vaccines among Japanese male patients who visited our urological clinics.

## 2. Materials and Methods 

### 2.1. Ethical Issues

All protocols and assessments in this study were approved by the ethics committees of Asoka Hospital (ID: 00008) and Nagareyama Central Hospital (ID: 00001) in Tokyo. Written informed consent was obtained from all participants in this study.

### 2.2. Sample Collection

The method of sample collection was reported previously [9]. In brief, we recruited 872 male patients aged 20 to 94 years who visited our urological clinics (Asoka Hospital and Nagareyama Central Hospital) from September 2011 to October 2015. Scraping samples from the glans penis were collected by the urologists with a wet cotton swab at least twice. For patients with phimosis, the urologists inserted a wet cotton swab inside the foreskin to collect scraping samples.

### 2.3. Questionaieres

The questionnaires included their age, educational status, smoking status, sexual orientation, number of lifetime sex partners, age at sex initiation, marital status, history of divorce, existence of current sex partners, and the presence or history of a sexually transmitted infection (STI).

### 2.4. Genotyping Analysis

One-mL aliquots of preservative solution containing scraping samples were centrifuged at 5000 rpm for 5 min, and the supernatant was discarded. The cell pellet was washed twice with 300 µL of 10 mmol/L Tris-HCl (pH 8.0). Deoxyribonucleic acid (DNA) was extracted from the cells using DNA extraction kit (SMI test, G & G Science Co., Fukushima, Japan). The genotype of HPV DNA and β-globin were assessed at the LSI Medience Corporation (Tokyo, Japan) using the GENOSEARCH HPV31 kit (Medical and Biological Laboratory, Nagoya, Japan) following the manufacturer’s instructions [10]. The assessment kit can detect the following 31 genotypes of HPV DNA: HR genotypes (16, 18, 31, 33, 35, 39, 45, 51, 52, 56, 58, 59, 68, 73, and 82) and LR genotypes (6b, 11, 26, 42, 44, 53, 54, 55, 61, 62, 66, 70, 71, 84, 90, and CP6108) by combination of multiplex polymerase chain reaction and Luminex technology [11,12]. It also assessed human β-globin to ensure that scraping samples from the glans penis contained enough cellular components.

### 2.5. Statistical Analysis

Statistical analyses in this study were performed using a JMP Pro software version 14 (SAS, Cary, NC, USA). A logistic regression analysis was used for detecting risk factors of HPV infection. The same control group (HPV negative plus β-globin positive cases) was used in all logistic regression analysis. *p* values < 0.05 were considered statistically significant.

## 3. Results and Discussion

Of the 872 scraping samples, β-globin was positive in 798 samples (91.5%). The characteristics of the participants, after excluding those who had negative β-globin are summarized in Table 1. The prevalence of HPV infection in our cohort is shown in Table 2. One hundred ninety-eight participants (198/798; 24.8%) had at least one type of any HPV infection. The total number of males with HPV infection targeted by 2v, 4v, and 9v were 20, 35, and 79, respectively.

**Figure 1:**
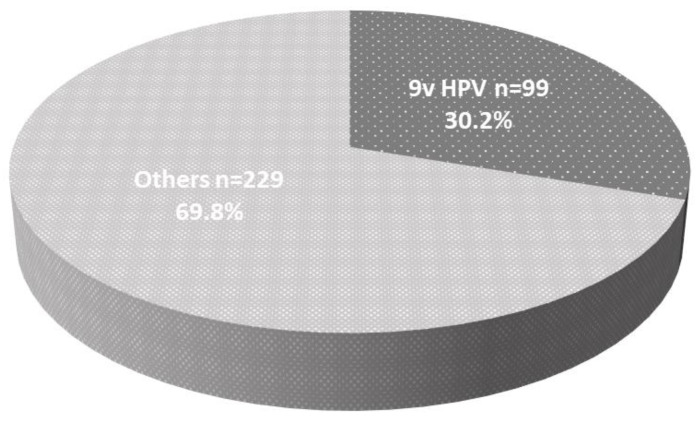
The detection rate of 9v human papilloma virus (HPV) among Japanese male sampling: Of 328 genotypes, 30.2% (99/328) were covered by the 9v HPV vaccine.

**Figure 2:**
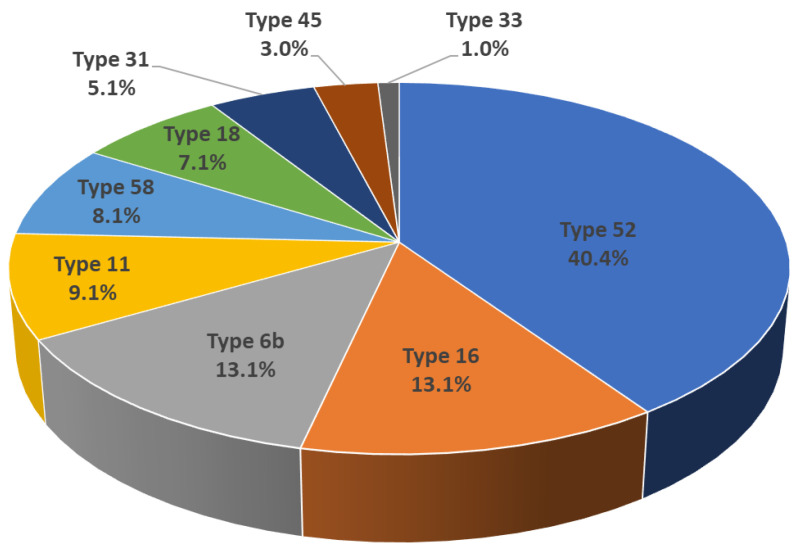
The detection rate among 9v HPVs: Type 52 was the most detected genotype (n = 40; 40/99; 40.4%).

The total number of HPV genotypes detected in this study was 328. Of the 328 HPV genotypes, 138 (42.1%) were HR HPV and 190 (57.9%) were LR HPV. The most frequently detected types of HR and LR HPV infections were type 52 (n = 40; 40/328; 12.2%) and type 90 (n = 26; 26/328; 7.9%), respectively. HPV genotypes 16, 18, 31, 33, 45, 52, and 58 were detected in 13, 7, 5, 1, 3, 40, and 8 participants, respectively. The number of HPV genotypes 6 and 11 were detected in 13 and 9 participants, respectively (three of the 19 participants were exposed to both types). Of the 19 participants exposed to HPV types 6b or 11, six participants (31.6%) suffered from condyloma acuminatum. Total number of detected HPV genotypes targeted by 9v HPV vaccine was 99 (99/328; 30.2%) (Figure 1). All the detected HPV genotypes and numbers are shown in Table 2; especially 9v HPV was drawn as a pie chart in Figure 2.

In 2013, Lenzi et al. reviewed five reports regarding the prevalence of HPV infection among patients attending the sexually transmitted disease clinic [13]. They reported that the prevalence of any HPV infection was 13.2% in Sweden [14], 44.9% in Denmark [15], 28.2% in the USA [16], 28.2% in the Netherlands [17], and 5.9% in Japan [18]. Regarding racial differences, Akogbe et al. reported that the prevalence of any HPV infection was significantly lower among Asians/Pacific Islander men (42.3%) compared to black (66.2%), Mexican (62.3%), and white (71.5%) men (*p* < 0.0001) [19]. Similar to the previous reports, the prevalence of any HPV infection among Japanese males in our study (24.8%) was relatively lower rather than other races because of fewer numbers of lifetime sexual partners. The most detected type of HPV among men was type 62 in Brazil (10.7%), type 59 in Mexico (7.2%), type 83 in the USA (7.2%) [20], and type 56 in Kenya (6.3%) [21]. According to the data of the British National Survey of Sexual Attitudes and Lifestyles, HPV DNA was detected in 17.4% and 29.0% of men and women, respectively [22]. In the study, the most detected HPV type was type 16 (2.3% and 4.2% of men and women, respectively). HPV type 52 was previously reported to be the most detected genotype (4.8%), which was also reported to be the most frequently detected genotype among young Japanese females [10,23]. In our study, HPV type 52 was the most frequently detected HR genotype among Japanese males.

In the multivariate logistic regression analysis, which included all cofounders that might interfere with each other, having ≥21 lifetime sex partners or a current or past STI were significant predictors against HPV infection with adjusted odds ratios (ORs) of 3.106 (95% confidence interval (CI), 1.593–6.059) and 1.894 (95% CI, 1.185–3.026), respectively (Table 3). Age of sex initiation (≤19 years) was a significant predictor against HPV infection targeted by 2v and 4v HPV vaccines with adjusted ORs of 5.100 (CI, 1.013–25.673; Table 4) and 2.676 (CI, 1.037–6.905; Table 5), respectively. Number of lifetime sex partners (≥21 persons) was a significant predictor against HPV infection targeted by 9v HPV vaccine with adjusted ORs of 2.397 (CI, 1.060–5.424; Table 6). Number of lifetime female sex partners was reported to be associated with HPV infection among male [24,25]. A younger age at first sexual intercourse also became a risk of HPV infection [25,26]. Smoking [26,27] was reported to aggravate risk of HPV infection; however, smoking was not associated with HPV infection in our cohort.

Since April 2013, HPV vaccinations have been recommended for girls aged 13 to 16 years in Japan; the aggressive recommendation of HPV vaccinations had been withheld for 10 weeks because adverse events, such as a long-term pain, numbness, and autoimmune diseases, had occurred in some vaccinated girls [28]. The efficacy and safety of HPV vaccination for Japanese males has been revealed in clinical trial recently [29]. Therefore, gender-neutral vaccination should be recommended in our society.

The strength of our study included a large sample size of adult males of all ages. However, we did not obtain information regarding whether the participants had any sexual contact with commercial sex workers, and both Asoka Hospital and Nagareyama Center Hospital locate in urban areas, which might be the weakness of our study.

In conclusion, we found that approximately a quarter of Japanese male patients who visited the urological clinics were exposed to HPV and that some kinds of sexual behavior aggravate the risk of typical HPV infections.

## 4. Conclusions

Approximately a quarter of Japanese male patients who visited urological clinics were exposed to at least one type of HPV infection, and some kinds of sexual behavior (such as number of lifetime sex partners, age of sex initiation, and present or past history of STIs) were found to be predictors for typical HPV infections.

## Figures and Tables

**Table 1:** Characteristics of study participants.

Characteristics	n OR Mean ± SD
Age, years	55.4 ± 19.5
Age group (years), n	
All	798
20–29	101
30–39	108
40–49	114
50–59	118
60–69	114
70–79	138
≥80	105
Educational status, n	
≤12 years	372
>12 years	412
Unknown	14
Smoking status, n	
Current	268
Previous	9
Never	503
Unknown	18
Sexual orientation, n	
Female	754
Male/Bisexual	13
Unknown	31
Number of lifetime sex partners, n	
Median (range)	5 (0 to 1500)
0 to 10	678
11 to 20	62
≥21	50
Unknown	8
Age of sex initiation, years	
Mean ± SD	20.8 ± 5.0
≤19	325
≥20	410
Unknown	63
Marital status, n	
Single/Never married	314
Married	464
Divorced	20
Sexual intercourse within a year, n (%)	
Yes	436
No	333
Unknown	29
Present or past history of sexually transmitted infections, n	
No	679
Yes	115
Unknown	4
Types of Sexually transmitted infection, n	
Condyloma acuminatum	11
Genital herpes	10
Syphilis	7
Gonococcal urethritis	58
Chlamydial urethritis	72

SD, standard deviation.

**Table 2:** The number of detected HPV genotypes among Japanese males.

Types of HPV	Number of Participants		
	Single HPV, n	Multiple HPVs, n	Total, n
**High-Risk HPV**			
Type 16 ^a,b,c^	3	10	13
Type 18 ^a,b,c^	1	6	7
Type 31 ^c^	0	5	5
Type 33 ^c^	0	1	1
Type 35	1	3	4
Type 39	6	7	13
Type 45 ^c^	1	2	3
Type 51	5	10	15
Type 52 ^c^	17	23	40
Type 56	6	6	12
Type 58 ^c^	3	5	8
Type 59	3	1	4
Type 68	5	4	9
Type 73	1	0	1
Type 82	2	1	3
**Low-risk HPV**			
Type 6b ^b,c^	5	8	13
Type 11 ^b,c^	3	6	9
Type 26	0	1	1
Type 42	5	9	14
Type 44	3	4	7
Type 53	6	11	17
Type 54	3	1	4
Type 55	1	5	6
Type 61	4	9	13
Type 62	8	11	19
Type 66	2	11	13
Type 70	1	7	8
Type 71	11	12	23
Type 84	3	7	10
Type 90	13	13	26
Type CP6108	4	3	7

Single HPV: Only one genotype was detected in one participant. Multiple HPVs: More than two genotypes were detected in one participant. The genotype targeted by ^a^ 2v, ^b^ 4v, and ^c^ 9v.

**Table 3:** Predictors against any HPV infection.

Variables	Crude OR (95% CI)	Adjusted OR * (95% CI)
Age, continuous	1.010 (1.002–1.019)	1.004 (0.991–1.017)
Educational status		
≤12 years	Reference	Reference
12 years	0.948 (0.685–1.311)	0.914 (0.627–1.334)
Smoking status		
Never	Reference	Reference
Current	1.670 ** (1.195–2.333)	1.091 (0.742–1.604)
Former	0.457 (0.057–3.695)	0.522 (0.060–4.164)
Sexual orientation		
Female	Reference	Reference
Male/Bisexual	1.929 (0.623–5.969)	1.372 (0.382–4.917)
Number of lifetime sex partners		
0 to 10	Reference	Reference
11 to 20	2.058 ** (1.185–3.574)	1.343 (0.736–2.453)
≥21	5.167 ** (2.861–9.331)	3.106 ** (1.593–6.059)
Age of sex initiation		
≥20 years	Reference	Reference
<19 years	2.114 ** (1.510–2.960)	1.480 (0.996–2.201)
Marital status		
Single/Never married	Reference	Reference
Married	1.180 (0.843-1.653)	1.231 (0.820–1.848)
Divorced	2.282 (0.898–5.799)	2.162 (0.780–5.992)
Sexual intercourse within a year, n		
No	Reference	Reference
Yes	1.282 (0.926–1.784)	1.295 (0.821–2.042)
Present or past history of STIs		
No	Reference	Reference
Yes	2.799 ** (1.860–4.197)	1.894 ** (1.185–3.026)

STI, sexually transmitted infection; OR, odds ratio; CI, confidence interval. * Multivariate analysis was performed with all variables. ** OR had significant differences (*p* < 0.05).

**Table 4:** Predictors against HPV infection targeted by the 2v HPV vaccine.

Variables	Crude OR (95% CI)	Adjusted OR * (95% CI)
Age, continuous	1.011 (0.988–1.034)	0.995 (0.957–1.036)
Educational status		
≥12 years	Reference	Reference
<12 years	1.236 (0.497–3.078)	1.155 (0.376–3.550)
Smoking status		
Never	Reference	Reference
Current	0.659 (0.257–1.691)	0.768 (0.254–2.327)
Former	NA	NA
Sexual orientation		
Female	Reference	Reference
Male/Bisexual	0.276 (0.034–2.251)	0.241 (0.023–2.567)
Number of lifetime sex partners		
0 to 10	Reference	Reference
11 to 20	0.239 ** (0.074–0.774)	0.384 (0.103–1.427)
≥21	0.258 ** (0.70–0.958)	0.654 (0.138–3.094)
Age of sex initiation		
≥20 years	Reference	Reference
<19 years	4.569 ** (1.489–14.017)	5.100 ** (1.013–25.673)
Marital status		
Single/Never married	Reference	Reference
Married	1.215 (0.498–2.968)	0.865 (0.268–2.792)
Divorced	NA	NA
Sexual intercourse within a year, n		
No	Reference	Reference
Yes	0.496 (0.175–1.405)	0.829 (0.177–3.872)
Present or past history of STIs		
No	Reference	Reference
Yes	0.382 (0.143–1.017)	0.418 (0.127–1.381)

STI, sexually transmitted infection; OR, odds ratio; CI, confidence interval; NA, not available. * Multivariate analysis was performed with all variables. ** OR had significant differences (*p* < 0.05).

**Table 5:** Predictors against HPV infection targeted by the 4v HPV vaccine.

Variables	Crude OR (95% CI)	Adjusted OR * (95% CI)
Age, continuous	0.981 (0.963–0.998)	0.987 (0.959–1.016)
Educational status		
≤12 years	Reference	Reference
>12 years	1.016 (0.511–2.024)	1.108 (0.483–2.544)
Smoking status		
Never	Reference	Reference
Current	1.815 (0.902–3.653)	1.553 (0.688–3.508)
Former	NA	NA
Sexual orientation		
Female	Reference	Reference
Male/Bisexual	1.880 (0.237–14.91)	1.906 (0.194–18.73)
Number of lifetime sex partners		
0 to 10	Reference	Reference
11 to 20	2.390 (0.879–6.503)	1.396 (0.463–4.216)
≥21	2.370 (0.789–7.118)	1.125 (0.305–4.159)
Age of sex initiation		
≥20 years	Reference	Reference
<19 years	3.393 (1.548–7.439)	2.676 ** (1.037–6.905)
Marital status		
Single/Never married	Reference	Reference
Married	2.234 ** (1.087–4.593)	2.190 (0.895–5.360)
Divorced	0.365 (0.098-1.356)	NA
Sexual intercourse within a year, n		
No	Reference	Reference
Yes	1.556 (0.743–3.255)	1.081 (0.392–2.982)
Present or past history of STIs		
No	Reference	Reference
Yes	2.491 ** (1.163–5.337)	2.136 (0.860–5.304)

STI, sexually transmitted infection; OR, odds ratio; CI, confidence interval; NA, not available. * Multivariate analysis was performed with all variables. ** OR had significant differences (*p* < 0.05).

**Table 6:** Predictors against HPV infection targeted by the 9v HPV vaccine.

Variables	Crude OR (95% CI)	Adjusted OR * (95% CI)
Age, continuous	0.984 (0.972–0.996)	0.986 (0.967–1.005)
Educational status		
≤12 years	Reference	Reference
>12 years	0.817 (0.508–1.316)	0.752 (0.434–1.303)
Smoking status		
Never	Reference	Reference
Current	1.796 (1.115–2.894)	1.148 (0.659–1.999)
Former	NA	NA
Sexual orientation		
Female	Reference	Reference
Male/Bisexual	1.722 (0.374–7.922)	1.215 (0.222-6.644)
Number of lifetime sex partners		
0 to 10	Reference	Reference
11 to 20	2.315 ** (1.112–4.821)	1.432 (0.645–3.181)
≥21	4.230 ** (2.117–8.453)	2.397 ** (1.060–5.424)
Age of sex initiation		
≥20 years	Reference	Reference
<19 years	2.669 ** (1.607–4.433)	1.590 (0.876–2.890)
Marital status		
Single/Never married	Reference	Reference
Married	0.798 (0.494–1.289)	0.987 (0.550–1.771)
Divorced	2.059 (0.651–6.515)	2.131 (0.600–7.565)
Sexual intercourse within a year, n		
No	Reference	Reference
Yes	2.166 ** (1.268–3.700)	1.243 (0.617–2.505)
Present or past history of STIs		
No	Reference	Reference
Yes	2.581 ** (1.507–4.421)	1.788 (0.960–3.329)

STI, sexually transmitted infection; OR, odds ratio; CI, confidence interval; NA, not available. * Multivariate analysis was performed with all variables. ** ORs had significant differences (*p* < 0.05).
